# Deciphering Risperidone-Induced Lipogenesis by Network Pharmacology and Molecular Validation

**DOI:** 10.3389/fpsyt.2022.870742

**Published:** 2022-04-18

**Authors:** Yun Fu, Ke Yang, Yepei Huang, Yuan Zhang, Shen Li, Wei-Dong Li

**Affiliations:** ^1^Department of Genetics, College of Basic Medical Sciences, Tianjin Medical University, Tianjin, China; ^2^Department of Psychiatry and Psychology, College of Basic Medical Sciences, Tianjin Medical University, Tianjin, China

**Keywords:** risperidone, lipogenesis, network pharmacology, molecular docking, lipolysis, beta oxidation

## Abstract

**Background:**

Risperidone is an atypical antipsychotic that can cause substantial weight gain. The pharmacological targets and molecular mechanisms related to risperidone-induced lipogenesis (RIL) remain to be elucidated. Therefore, network pharmacology and further experimental validation were undertaken to explore the action mechanisms of RIL.

**Methods:**

RILs were systematically analyzed by integrating multiple databases through integrated network pharmacology, transcriptomics, molecular docking, and molecular experiment analysis. The potential signaling pathways for RIL were identified and experimentally validated using gene ontology (GO) enrichment and Kyoto encyclopedia of genes and genomes (KEGG) analysis.

**Results:**

Risperidone promotes adipocyte differentiation and lipid accumulation through Oil Red O staining and reverse transcription-polymerase chain reaction (RT-PCR). After network pharmacology and GO analysis, risperidone was found to influence cellular metabolism. In addition, risperidone influences adipocyte metabolism, differentiation, and lipid accumulation-related functions through transcriptome analysis. Intersecting analysis, molecular docking, and pathway validation analysis showed that risperidone influences the adipocytokine signaling pathway by targeting MAPK14 (mitogen-activated protein kinase 14), MAPK8 (mitogen-activated protein kinase 8), and RXRA (retinoic acid receptor RXR-alpha), thereby inhibiting long-chain fatty acid β-oxidation by decreasing STAT3 (signal transducer and activator of transcription 3) expression and phosphorylation.

**Conclusion:**

Risperidone increases adipocyte lipid accumulation by plausibly inhibiting long-chain fatty acid β-oxidation through targeting MAPK14 and MAPK8.

## Introduction

Antipsychotic drugs are the cornerstone of the current treatment of schizophrenia. In addition, antipsychotics can be used to treat other mental disorders, including bipolar disorder, autism spectrum disorder, obsessive-compulsive disorder, and dementia (1–3). Compared with first-generation antipsychotics (FGAs), second-generation antipsychotics (SGAs) have shown better improvements in adherence, cognitive function, negative symptoms, and dyskinesia, making them first-line clinical agents (4). However, the administration of SGA could generate several reported adverse effects, such as weight gain, obesity, metabolic disturbances, and hepatic and renal dysfunctions (5–7). These side effects are high-risk factors for diabetes, cardiovascular disease, and cerebrovascular disease (8–10), leading to poor treatment compliance.

Unlike other SGAs, such as clozapine and olanzapine, risperidone is a derivative of benzisoxazole with multiple receptor antagonist properties, making it more effective against negative symptoms and more beneficial on both affective symptoms and cognitive impairments (11). Risperidone-induced weight gain is associated with a number of factors, including gene polymorphisms (12–14), exercise (15–17), peripheral molecules (18), and hyperphagia caused by regulating the expression of melanocortin-4 receptor (MC4R), neuropeptide Y (NPY), and agouti-related peptide (AgRP) (19–21). It is also related to increased adiponectin (APN) expression associated with adipocyte differentiation, as well as the expression of adipogenic genes such as peroxisome proliferator-activated receptor (*PPAR)* (22, 23). Risperidone upregulates fatty acid synthase (FASN) and sterol regulatory element-binding protein 1 (SREBP1) expression in hepatocyte cultures and mouse liver by targeting the hepatic SREBP-1c/FASN couple, which is also one of the mechanisms by which risperidone induces weight gain (24). Interestingly, some studies have linked risperidone-induced weight gain with lipolysis and considered that risperidone increases lipid accumulation by altering lipolysis (25). Risperidone inhibits leptin-mediated phosphorylation of STAT3-Y705, leading to weight gain (26). Our previous work and other studies have observed that risperidone promotes lipid accumulation (24, 27). However, no studies have comprehensively explained the drug targets and molecular mechanisms.

Network pharmacology is commonly used to predict drug-binding targets to explore the mechanisms of drug action and the induction of drug side effects (28, 29). In contrast to traditional molecular mechanistic studies, network pharmacology provides systems-level insights into drug-disease interactions due to its “multi-gene, multi-target” nature. Providing a detailed drug-target-pathway network helps to assess the rationality and compatibility of drugs. At present, it has been widely used in the study of drug mechanisms for the treatment of Alzheimer's disease, anxiety, and other mental diseases (30, 31).

**Figure 1:**
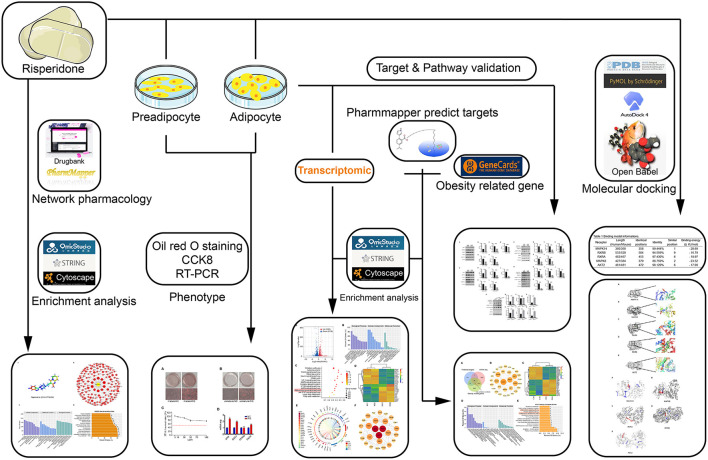
Schematic of the workflow.

To the best of our knowledge, no study has clarified the mechanisms of risperidone-induced weight gain using network pharmacology and molecular docking. Here, in combination with transcriptomic sequencing, network pharmacological target analysis, and molecular mechanisms, we aimed to investigate the possible mechanisms of risperidone-induced lipid accumulation through the fatty acid β-oxidation pathway from the perspective of lipid decomposition, which may provide a new understanding and strategy for risperidone-induced weight gain. A detailed schematic diagram of the workflow of this study is shown in Figure 1.

## Materials and Methods

### Cell Culture and Treatment

3T3-L1 embryonic fibroblasts (purchased from BeNa Culture Collection, China) and adipose tissue mesenchymal stem cells (AMSCs) [isolated as previously described by Liu et al. (32)] are commonly used models to study adipocyte differentiation *in vitro* for performing this experiment. Briefly, inguinal adipose tissue dissected from 6-week-old mice (C57BL/6J) was washed three times in PBS containing 2% of penicillin-streptomycin (Cat# 15070063, GIBCO, California, USA) and cut into fragments. Then, the fragments were digested into single cells using collagenase 1 (Cat# A004194, Sangon Biotech, China) for 1 h and finally cultured for ~4 days in Dulbecco's Modified Eagle Medium (DMEM, Cat# SH30022.01, HyClone, Utah, USA) containing 20% of fetal bovine serum (Cat# 11011-8611, EveryGreen, China), 1% of l-glutamine (Cat# 56-8-59, Sigma, Missouri, USA), and 1% of penicillin-streptomycin. AMSC and 3T3-L1 cells were cultured with DMEM containing 10% of fetal bovine serum (FBS) and 1% of penicillin-streptomycin at 37°C in 5% of CO_2_.

Preadipocyte 3T3-L1 cells and AMSCs were differentiated as previously described by Hilgendorf et al. (33). Briefly, AMSC and 3T3L1 cells were sequentially treated with MDI (0.5 mM isobutyl-methylxanthine (Cat# I8450, Solarbio, China), 1 μM of dexamethasone (Cat# D8040, Solarbio, China), and 10 ng/L of insulin (Cat# 11070-73-8, Sigma, Missouri, USA) for 2 days, 10 ng/L of insulin for another 2 days, and 2.5 ng/L of insulin for the last 2 days. For drug treatment, differentiated cells were treated with dimethyl sulfoxide (DMSO, Cat# D8370, Solarbio, China) and risperidone (Cat# HY-11018, MCE, China) for 48 h. In addition, we used undifferentiated cells treated with the same drugs as differentiated cells to explore the effect of risperidone on differentiation.

### Cell Viability Assay

The effect of risperidone on cell viability in 3T3-L1 preadipocytes was assessed using a Cell Counting Kit-8 (CCK-8, Cat# BS350B, Biosharp, China). Briefly, 5,000 cells were seeded in a 96-well plate overnight and treated with gradient concentrations (0, 10, 30, 50, 70, and 100 μM) of risperidone for 48 h. Then, 10 μl of CCK-8 solution was added to each well and incubated for 1–4 h. The results were measured by a microplate reader (MULTISCKAN GO, Thermo Fisher Scientific, Rockford, IL, USA) under a 450-nm extraction laser. Each set contained three replicates.

### Oil Red O Staining

The Oil Red O (ORO, Cat# O0625, Sigma, Missouri, America) solution was prepared by dissolving 0.3 g of ORO powder in 60 ml of isopropanol and diluting with 40 ml of distilled water. Before staining, the solution was filtered through a filter to obtain a clear solution. The cells were carefully washed twice with PBS and fixed with 4% of paraformaldehyde for 20 min. Then, the cells were rinsed with PBS and stained with ORO solution for 20 min at room temperature (RT). Finally, the cells were washed 2–5 times with distilled water until there was no excess ORO staining solution. The cells were observed and photographed under an Olympus IX71 inverted microscope (Olympus, Japan).

### cDNA Synthesis and qRT-PCR Analysis

Total RNA from control and risperidone-treated adipocytes was extracted with TRIzol reagent (Cat# 260802, Life, New York, USA) according to the instructions. RNA quality and concentration were measured using a microplate reader at 260/280 nm. Total RNA (1 μg) from each sample was reverse transcribed into complementary cDNA using a cDNA Synthesis Kit (Cat# B24408, Bimake, China). After synthesis, cDNA was diluted 10 times and subjected to qRT-PCR amplification using an SYBR Green Real-Time PCR Master Mix (Cat# B21703, Bimake, China) and gene-specific primer pairs (Adiponectin: Forward: GGACTCTACTACTTCTCTTACC and Reverse: CAGATGGAGGAGCACAGA; RAC1: Forward: TGTAGCCGTATTCATTGTCA and Reverse: GTCGCACTTCAGGATACC; PPARg: Forward: TTATGGGTGAAACTCTGGGA and Reverse: AATCAACTGTGGTAAAGGGC; and FASN: Forward: GCCCGGTAGCTCTGGGTGTA and Reverse: TGCTCCCAGCTGCAGGC). The mRNA expression was normalized to 18S rRNA and analyzed using the 2^−Δ*ΔCt*^ method.

### Transcriptome Sequencing and Analysis

Two replicated transcriptome libraries were prepared from DMSO- and RIS-treated differentiated 3T3-L1 cells. A total of four libraries were sequenced using the MGISEQ-2000 sequencer (BGI, Shenzhen, China). For each RNA sample, cells were collected from three replicates and pooled after RNA extraction. Raw sequencing reads were cleaned by removing adaptor sequences, reads containing poly-N sequences, and low-quality reads. After mapping the data, normalization was performed, and fragments per kilobase million (FPKM) mapped reads were calculated using the DC.TOM platform. Raw RNA sequencing data were deposited at the GEO repository (GSE198053, https://www.ncbi.nlm.nih.gov/geo/query/acc.cgi?acc=GSE198053). The significance levels of terms and pathways were corrected with a rigorous threshold by Bonferroni, shown as the Q value (≤ 0.05).

### Network Pharmacology

The structure of risperidone (Drug Bank ID: DB00734) was downloaded from DrugBank (https://go.drugbank.com/) in PDB format and converted to mol2 format using Open Babel 2.4.1 software. The potential targets of risperidone were predicted by PharmMapper (http://www.lilab-ecust.cn/pharmmapper/) using the Human Protein Targets Database (34).

### The Construction of Visualization Networks

The obesity-related genes were obtained from the GeneCards database (https://www.genecards.org/) by the keyword “obesity.” The cross-targets of risperidone-, transcriptome-, and obesity-related genes were queried in the STRING database (version 11.0) to obtain the interaction network (confidence ≥0.4) and the enrichment results of high-confidence candidates using Cytoscape 3.9.1 software for visual optimization of the protein-protein interaction (PPI) results. In addition, the gene ontology (GO) and Kyoto encyclopedia of genes and genomes (KEGG) enrichments were visualized using OmicStudio (https://www.omicstudio.cn/tool), supported by the R language.

### Molecular Docking

The binding sites and interaction forces for proteins and small molecules can be predicted using molecular docking analysis. First, the molecular structure of risperidone was obtained from DrugBank (https://go.drugbank.com/) in PDB format, and the protein structures of predicted targets (MAPK14, MAPK8, RXRA, RXRB [retinoic acid receptor RXR-β], and AKT2 [RAC-β serine/threonine-protein kinase]) were obtained from the PDB database in PDB format (https://www.rcsb.org/). All water and ligands were removed using PyMOL version 1.2R2 and saved in PDBQT format for molecular docking, so our predictions were performed under nonaqueous conditions. Next, we evaluated the predicted posture of the interactions between small molecules and predicted targets for network pharmacology using the AutoDock 4.2 package. The molecular docking mode we used was semiflexible, and the analysis settings for the grid box were set as a cube of 62 × 56 × 64, 40 × 40 × 70, 60 × 52 × 68, 88 × 56 × 88, and 60 × 90 × 50 (x, y, z) with 1.000 Å and centered at the grid point of receptors MAPK14, MAPK8, RXRA, RXRB, and AKT2, respectively. The models were visualized using PyMOL 1.2R2 version.

### Protein Extraction and Western Blot Analysis

Cells were washed 3 times with ice-cold PBS, lysed with RIPA lysis buffer (Cat# E1013+, Applygen, China) containing protease inhibitors (Cat# P1265, Applygen, China), phosphorylase inhibitor (Cat# B15000, Bimake, China) and PMSF (Cat# P0100, Solarbio, China) for 20 min, centrifuged at 12,000 rpm for 20 min at 4°C, and then boiled with loading buffer (Cat# E153-01, Genstar, China). Each sample was separated by SDS-PAGE (10%) gel (Cat# E153, Genstar, China) and transferred into a piece of polyvinylidene fluoride (PVDF, Cat# IPV00010, Merck Millipore, MA, USA) membrane. Nonspecific binding was blocked by soaking the membrane in Tris-buffered saline-Tween (TBST) buffer that contained 5% of nonfat dry milk (Cat# A600669-0250, Sangon Biotech, China) or bovine serum albumin (BSA, Cat# C508113-0001, Sangon Biotech, China) for 2 h at RT. The membrane was incubated overnight with the primary antibody at 4°C, including anti-P-mapk14-T180/Y182 antibody (AP0526, 1:1,000 dilution, Abclonal, China), anti-mapk14 antibody (A0227, 1:1,000 dilution, Abclonal, China), anti-P-stat3-Y705 antibody (AP0705, 1:1,000 dilution, Abclonal, China), anti-P-stat3-S727 antibody (AP0715, 1:1,000 dilution, Abclonal, China), anti-stat3 antibody (A19566, 1:1,000 dilution, Abclonal, China), anti-cpt1A antibody (A20746, 1:1,000 dilution, Abclonal, China), anti-tubulin-β antibody (AF7011, 1:3,000 dilution, Affinity, USA), anti-mapk8 antibody (A2462, 1:1000 dilution, Abclonal, China), anti-rxrB antibody (A18119, 1:1,000 dilution, Abclonal, China), anti-rxrA antibody (A19015, 1:1,000 dilution, Abclonal, China), anti-akt2 antibody (A18019, 1:1,000 dilution, Abclonal, China), anti-actin-β antibody (A026, 1:25,000 dilution, Abclonal, China), and anti-ppar-gamma antibody (A0270, 1:1,000 dilution, Abclonal, China). After washing with TBST buffer, the membrane was incubated with goat anti-rabbit (Cat# S0001, Affinity, USA) or goat anti-mouse HRP-linked secondary antibody (Cat# S0002, Affinity, USA) at a dilution of 1:10,000 for 2 h at RT. Chemiluminescence solution (Cat# 201005-79, Advansta, California, USA) and medical X film (Carestream, China) were used for detection, and ImageJ software was used for analysis.

### Statistical Analysis

Statistical analyses and figures were generated using GraphPad Prism 9.0 (GraphPad Software Inc., CA, USA). The values in the figures are presented as the mean ± standard error of the mean (SEM). A *p*-value of <0.05 was considered statistically significant.

## Results

### Risperidone Promotes Lipid Accumulation in Both Undifferentiated and Differentiated Adipocytes

**Figure 2:**
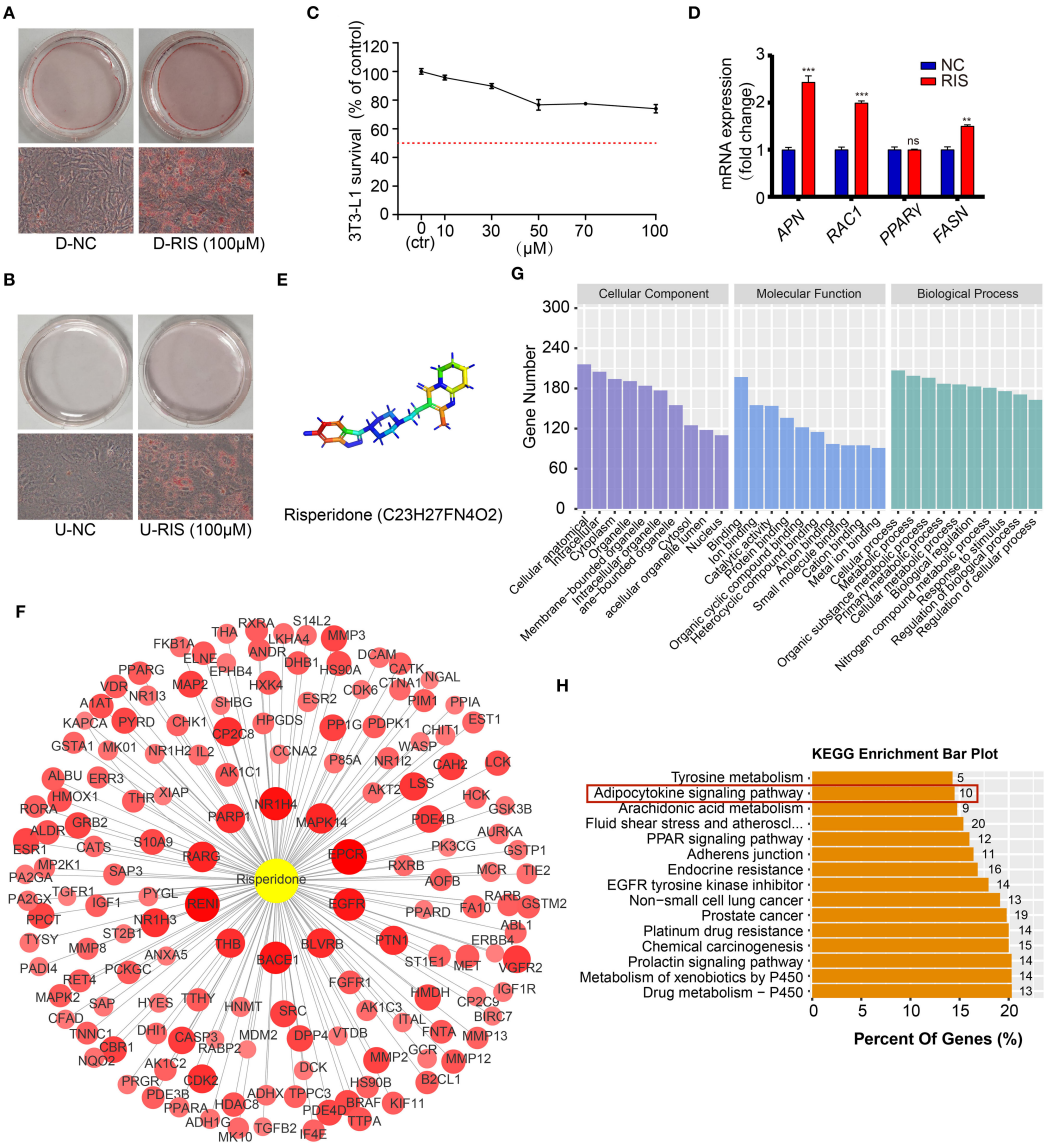
Risperidone promotes adipogenesis and network pharmacology analysis. Oil red O staining of **(A)** differentiated 3T3-L1 cells and **(B)** undifferentiated 3T3-L1 cells treated with DMSO or risperidone. **(C)** Risperidone toxicity tested by cell counting kit 8 (CCK8). **(D)** The mRNA levels of genes related to adipocyte differentiation and lipid accumulation in differentiated 3T3-L1 cells treated with DMSO or risperidone measured by qRT-PCR. **(E)** Structure of risperidone. **(F)** Predicted target proteins of risperidone. Bubble size and depth of color are proportional to the *Z-*score (Z > 0). **(G)** GO enrichment analysis. **(H)** KEGG enrichment analysis of predicted targets. D-NC, differentiated 3T3-L1 cells treated with DMSO; D-RIS, differentiated 3T3-L1 cells treated with risperidone; U-NC, undifferentiated 3T3-L1 cells treated with DMSO; U-RIS, undifferentiated 3T3-L1 treated with risperidone. **P < 0.01, ***P < 0.005.

To test whether risperidone could directly induce weight gain by promoting lipid accumulation in adipocytes and preadipocytes, differentiated and undifferentiated cells (3T3-L1 and AMSC) were exposed to risperidone for 48 h. After ORO staining, risperidone promoted adipocyte lipid accumulation and preadipocyte differentiation (Figures 2A,B and Supplementary Figures 1A,B). As shown in Figure 2C, risperidone inhibited the growth of 3T3L1 preadipocytes in a dose-dependent manner. Risperidone (100 μM) had little effect on the cell survival of 3T3L1 preadipocytes; therefore, 100 μM of risperidone was used in the following *in vitro* experiments. We also detected the transcript levels of adipocyte-related genes (Figure 2D) and found that risperidone promoted the expression of *Apn, Rac1* (Rac family small GTPase 1), and *Fasn*. Taken together, risperidone promoted lipid accumulation in undifferentiated and differentiated 3T3-L1 cells and AMSCs.

### Prediction and Analysis of Risperidone Binding Targets

To explore the mode of action of risperidone, the targets of risperidone were predicted using network pharmacology analysis. We obtained the structure of risperidone from the Drug Bank (Figure 2E) and collected 236 predicted targets by network pharmacology analysis (Supplementary Table 1). The 153 targets that met the criterion of Z > 0 were considered strongly correlated targets (Figure 2F) for further analysis. GO enrichment analysis indicated that risperidone affected a series of biological processes, including cellular processes, metabolic processes, organic substance metabolic processes, primary metabolic processes, cellular metabolic processes, biological regulation, nitrogen compound metabolic processes, responses to stimuli, regulation of biological processes, and regulation of cellular processes (Figure 2G). In addition, in the KEGG pathway analysis, 155 KEGG pathways were selected (*P*-adjust < 0.05) related to all predicted targets, including the drug metabolism-cytochrome P450, metabolism of xenobiotics by cytochrome P450, prolactin signaling pathway, chemical carcinogenesis, platinum drug resistance, prostate cancer, non-small cell lung cancer, EGFR tyrosine kinase inhibitors, adherens junctions, PPAR signaling pathway, fluid shear stress and atherosclerosis, arachidonic acid metabolism, adipocytokine signaling pathway, and tyrosine metabolism (Figure 2H).

### Risperidone Influenced Metabolic and Lipid β-Oxidation in Adipocytes

**Figure 3:**
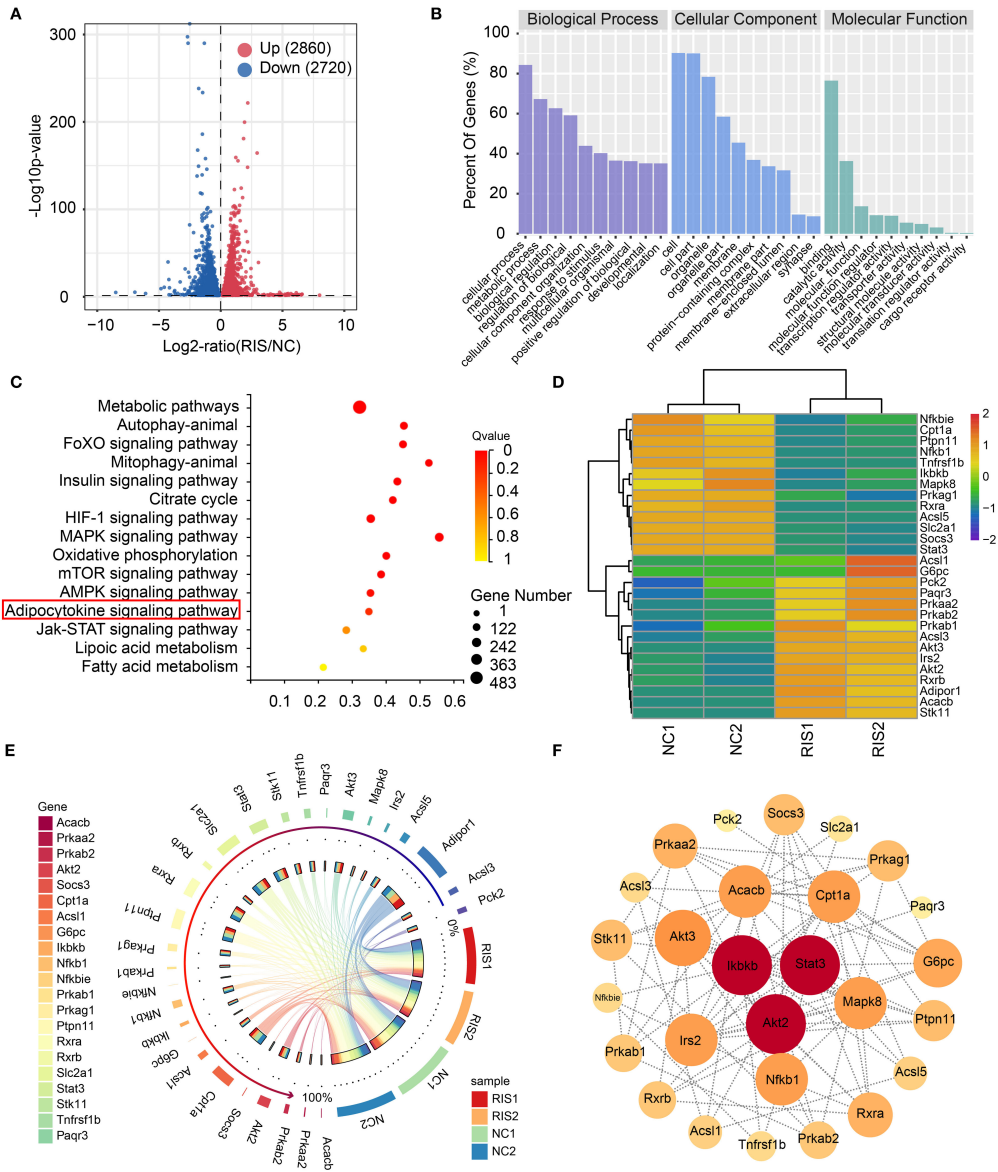
Transcriptome sequencing analysis showed that risperidone influenced cell metabolism and the adipocytokine signaling pathway. **(A)** Volcano plot showing gene transcription influenced by risperidone in differentiated 3T3-L1 cells (*P* < 0.05, *n* = 2). Upregulated genes are shown in the right panel (red circles, 2,080 genes), and downregulated genes are shown in the left panel (blue circles, 2,720 genes). **(B)** GO enrichment analysis. **(C)** KEGG enrichment analysis of genes influenced by risperidone shown in **(A)**. **(D)** Heat map of risperidone-influenced genes in the adipocytokine signaling pathway. **(E)** Expression circos map of risperidone-influenced genes in the adipocytokine signaling pathway. **(F)** Interaction network of risperidone-influenced genes in the adipocytokine signaling pathway. The bubble size and depth of color are proportional to the node degree. NC1 and NC2 were control samples, and RIS1 and RIS2 were risperidone-treated samples.

To determine the molecular mechanisms underlying lipid accumulation in response to risperidone treatment, differentiated 3T3-L1 cells treated with risperidone or DMSO were tested by transcriptome sequencing (mRNA-seq, *n* = 2). Compared with controls, risperidone caused the upregulation of 2,860 genes and the downregulation of 2,720 genes (Figure 3A). A total of 5,580 genes were used for GO and KEGG enrichment analysis, indicating that risperidone affected a range of biological processes, including cellular processes, metabolic processes, and biological regulation (Figure 3B). In the KEGG pathway analysis, risperidone affected the metabolic pathway, autophagy-animal, FoXO signaling pathway, mitophagy-animal, insulin signaling pathway, citrate cycle, HIF-1 signaling pathway, MAPK signaling pathway, oxidative phosphorylation, mTOR signaling pathway, AMPK signaling pathway, adipocytokine signaling pathway, JAK-STAT signaling pathway, lipoic acid metabolism, and fatty acid metabolism (Figure 3C). Based on these results, we investigated whether the adipocytokine signaling pathway played an important role in risperidone-induced lipid accumulation. On the one hand, transcriptomic results showed that risperidone downregulated 13 genes, including *Nfkbie* (NFKB inhibitor epsilon), *Cpt1a* (carnitine palmitoyltransferase 1A), *Ptpn11* (protein tyrosine phosphatase non-receptor type 11), *Nfkb1* (nuclear factor kappa B subunit 1), *Tnfrsf1b* (TNF receptor superfamily member 1B), *Ikbkb* (inhibitor of nuclear factor kappa B kinase subunit β), *Mapk8, Prkag1* (protein kinase AMP-activated non-catalytic subunit gamma 1), *Rxra, Acsl5* (acyl-CoA synthetase long chain family member 5), *Slc2a1* (solute carrier family 2 member 1), *Socs3* (suppressor of cytokine signaling 3), and *Stat3*, and up-regulated 15 genes, including *Acsl1* (acyl-CoA synthetase long chain family member 1), *G6pc* (glucose-6-phosphatase, catalytic), *Pck2* (phosphoenolpyruvate carboxykinase 2), *Paqr3* (progestin and adipoQ receptor family member 3), *Prkaa2* (protein kinase AMP-activated catalytic subunit alpha 2), *Prkab2* (protein kinase AMP-activated non-catalytic subunit β 2), *Prkab1* (protein kinase AMP-activated non-catalytic subunit β 1), *Acsl3* (acyl-CoA synthetase long chain family member 3), *Akt3* (AKT serine/threonine kinase 3), *Akt2, Irs2* (insulin receptor substrate 2), *Rxrb, Adipor1* (adiponectin receptor 1), *Acacb* (acetyl-CoA carboxylase β), and *Stk11* (serine/threonine kinase 11) (Figure 3D). In addition, we found that the major highly expressed genes in the adipocytokine signaling pathway, such as *Cpt1a, Ptpn11, Rxra, Slc2a1, Stat3*, and *Prkag1*, were downregulated after risperidone treatment (Figure 3E). To explore the relationships among the core set genes and provide a global view of network architecture, we set up a PPI network analysis model using STRING online (combined score ≥ 0.4) and Cytoscape software. The top three proteins with relatively high connectivity degrees were STAT3 (node degree = 11), AKT2 (node degree = 11), and IKBKB (node degree = 11) (Figure 3F).

### Analysis of Intersecting Targets in Network Pharmacology/Transcriptomics/Obesity-Related Genes

**Figure 4:**
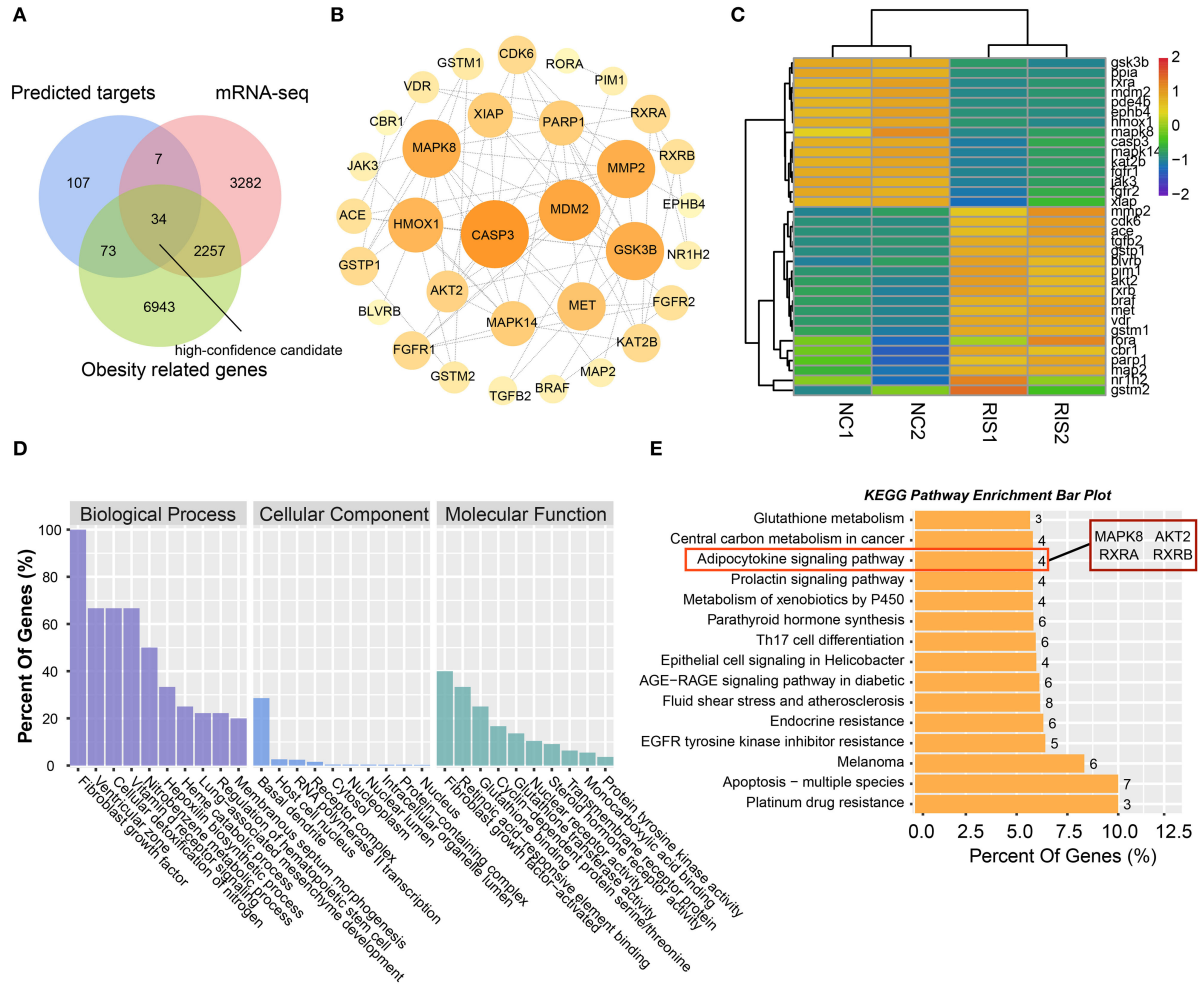
Analysis of intersected targets in network pharmacology/transcriptome/obesity-related genes. **(A)** Intersected targets or genes of risperidone and transcriptome- and obesity-related genes were considered high-confidence candidates. **(B)** Interaction network of high-confidence candidates described in **(A)**. **(C)** Heat map showing the expression level of high-confidence candidates in samples. **(D)** GO analysis. **(E)** KEGG analysis of high-confidence candidates.

To obtain high-confidence candidates, we first accessed 9,307 obesity-related genes from the GeneCards online database using the key word “obesity.” After comparing the predicted targets of risperidone, transcripts influenced by risperidone, obesity-related genes, and 34 intersecting targets were considered high-confidence candidates (Figure 4A). To explore the relationships among the 34 targets, we built a PPI network model. The top five proteins with higher degrees of connectivity were CASP3 (caspase-3) (node degree = 14), MDM2 (E3 ubiquitin-protein ligase mdm2) (node degree = 12), MAPK8 (node degree = 11), GSK3B (glycogen synthase kinase-3 β) (node degree = 11), and MMP2 (72 kDa type IV collagenase) (node degree = 11) (Figure 4B). Interestingly, transcriptomic sequencing showed that the top five targets of CASP3, MDM2, MAPK8, and GSK3B were downregulated (Figure 4C). Furthermore, GO and KEGG enrichment analyses of these 34 targets showed that risperidone affected biological processes, including the fibroblast growth factor receptor signaling pathway involved in orbitofrontal cortex development, ventricular zone neuroblast division, cellular detoxification of nitrogen compounds, the vitamin D receptor signaling pathway, and the nitrobenzene metabolic process (Figure 4D). In the KEGG analysis, risperidone affected platinum drug resistance, apoptosis-multiple species, EGFR tyrosine kinase inhibitor resistance, the AGE-RAGE signaling pathway in diabetic complications, metabolism of xenobiotics by cytochrome P450, adipocytokine signaling pathway, etc (Figure 4E).

### Molecular Docking for Risperidone and Immunoblot Validation

**Figure 5:**
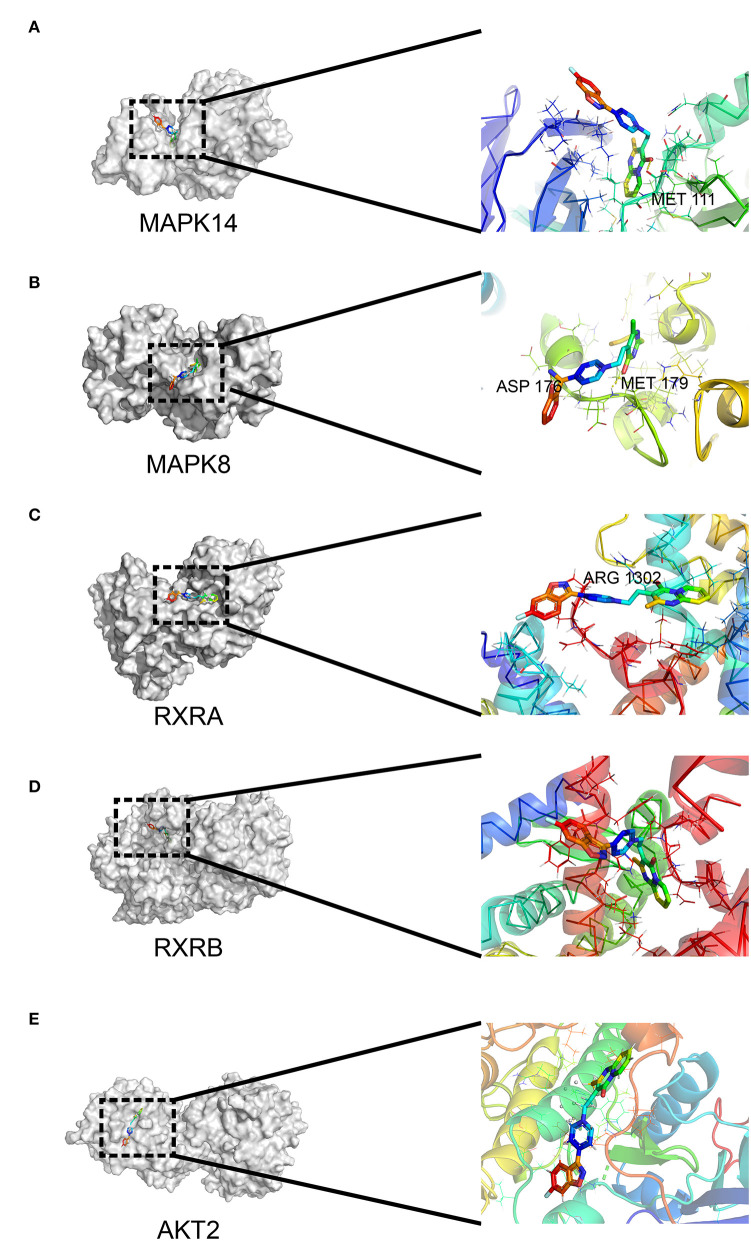
Interaction between risperidone and predicted targets in the adipocytokine signaling pathway using molecular docking analysis. Models of risperidone (DRUGBANK ID DB00734) with **(A)** mitogen-activated protein kinase 14 (MAPK14, PDB ID 6HWU), **(B)** mitogen-activated protein kinase 8 (MAPK8, PDB ID 4AWI), **(C)** retinoic acid receptor RXR-alpha (RXRA, PDB ID 7B9O), **(D)** retinoic acid receptor RXR-β (RXRB, PDB ID 1H9U), and **(E)** RAC-β serine/threonine-protein kinase AKT2 (PDB ID 3E87).

**Figure 6:**
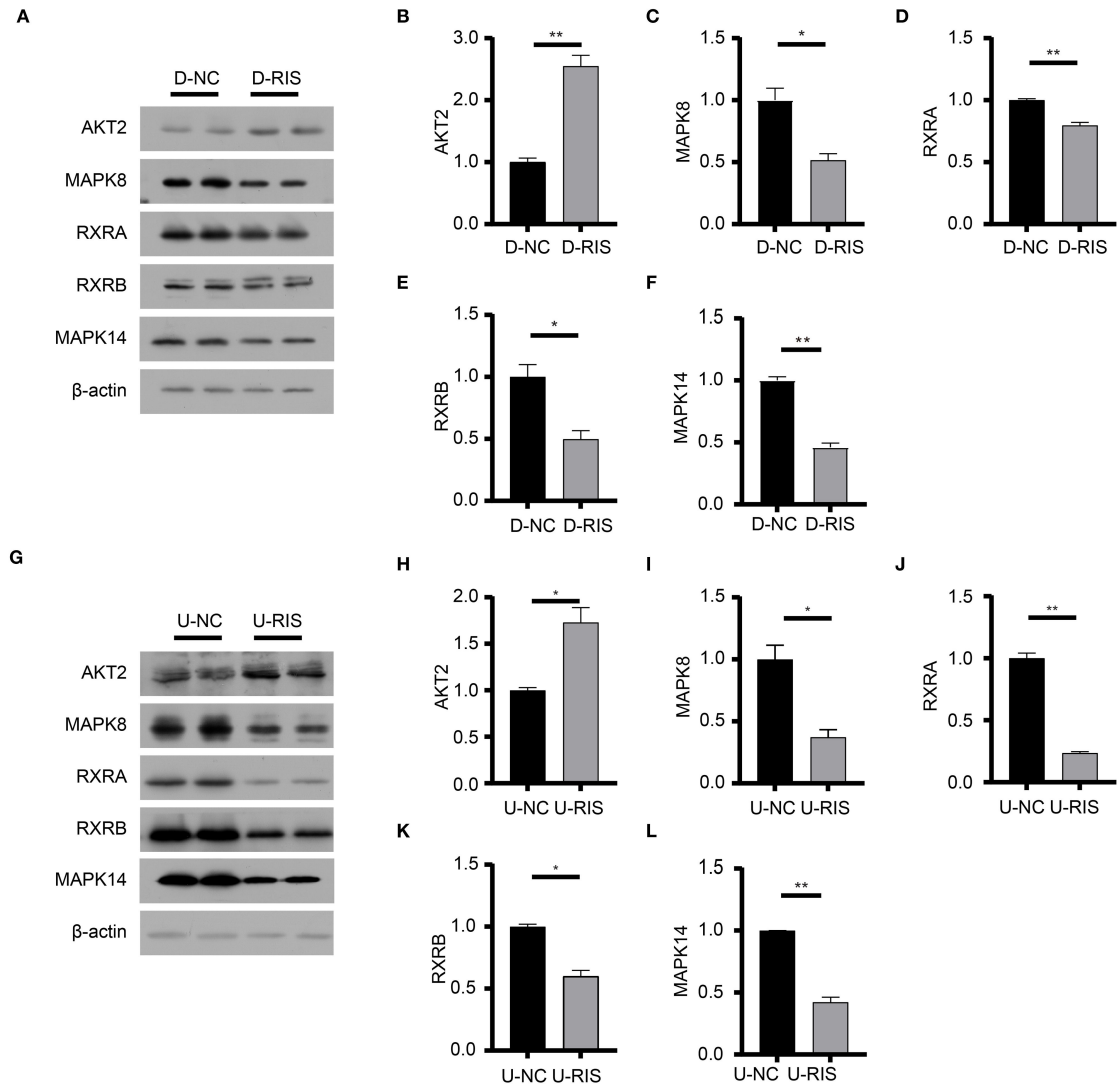
Immunoblotting analysis of molecular docking targets. **(A)** Expression level of the molecular docking targets in differentiated 3T3-L1 cells after treatment with DMSO or risperidone. **(B**–**F)** Gray degree analysis shows the expression level of targets in **(A)**. **(G)** Expression level of the molecular docking targets in undifferentiated 3T3-L1 cells after treatment with DMSO or risperidone. **(H**–**L)** Gray degree analysis shows the expression level of targets in **(G)**. Values are expressed as the mean ± SEM (*n* = 2). *P < 0.05, **P < 0.01; ns, no significant difference; SEM, standard error of mean; D-NC, differentiated 3T3-L1 cells treated with DMSO; D-RIS, differentiated 3T3-L1 cells treated with risperidone; U-NC, undifferentiated 3T3-L1 cells treated with DMSO; U-RIS, undifferentiated 3T3-L1 treated with risperidone.

To determine the potential binding of risperidone to intersecting targets, molecular docking analysis was performed. The adipocytokine signaling pathway was enriched in network pharmacology (Figure 2H) and transcriptome sequencing (Figure 3C), as well as obesity-related genes (Figure 4E). We focused on factors in the adipocytokine signaling pathway. As reported previously, the adipocytokine signaling pathway is closely related to lipid metabolism and obesity, so MAPK8, RXRA, RXRB, and AKT2, which are involved in the adipocytokine signaling pathway and were screened as intersecting targets in network pharmacology/transcriptomic/obesity-related genes, were considered candidate genes for molecular docking and verification. Moreover, we noticed that MAPK14 is a critical upstream gene that regulates adipocytokine signaling pathways and obtained a high Z-value in network pharmacological predictions (which indicates a target with promising binding capacity and functional importance). Therefore, MAPK14 was also considered a candidate and verified in molecular docking. The crystal structure of the intersecting targets was collected from the PDB database with PDB IDs 6HWU, 4AWI, 7B9O, and 1H9U for docking analysis with risperidone. The MAPK14 docking results showed that the hydrogen bond between risperidone and the MAPK14 protein acted on one amino acid residue, *viz*. MET-111 (Figure 5A). The MAPK8 docking results showed that the hydrogen bond between risperidone and the MAPK8 protein acted on two amino acid residues, namely, ASP-176 and MET-179 (Figure 5B). The RXRA docking results showed that the hydrogen bond between the risperidone and RXRA proteins acted on one amino acid residue, namely, ARG-1302 (Figure 5C). The RXRB docking results showed that there was no hydrogen bond between the risperidone and RXRB proteins (Figure 5D). The AKT2 docking results showed that there was no hydrogen bond between the risperidone and AKT2 proteins (Figure 5E). The minimal combinations of these models are shown in Table 1. Briefly, the minimum lowest binding energies of MAPK14, RXRB, RXRA, MAPK8, and AKT2 for risperidone were −20.69, −16.79, −18.97, −23.52, and −17.09 KJ/mol, respectively. Furthermore, we performed docking comparisons of risperidone and the binding position of ligands of the protein crystal (Supplementary Figures 2A–E), and we found a partial overlap between risperidone and ligands in MAPK14 (Supplementary Figure 2A) and MAPK8 (Supplementary Figure 2B), suggesting that risperidone may have a good binding activity to MAPK14 and MAPK8. To verify the effects of risperidone on molecular docking targets of MAPK14, MAPK8, RXRA, RXRB, and AKT2, immunoblotting analysis was performed on differentiated 3T3-L1 cells after treatment with DMSO or risperidone. The results showed that MAPK14, MAPK8, RXRA, and RXRB were downregulated in risperidone treatment, while the AKT2 was upregulated (Figures 6A–F). We also detected the expression levels of these molecular docking targets in undifferentiated 3T3-L1 cells and got similar results (Figures 6G–L).

**Table 1:** Binding model information.

**Receptors**	**Length (Human/Mouse)**	**Identical positions**	**Identity**	**Similar position**	**Binding energy (Δ KJ/mol)**
MAPK14	360/360	358	99.444%	1	−20.69
RXRB	533/520	504	94.559%	9	−16.79
RXRA	462/467	455	97.430%	6	−18.97
MAPK8	427/384	379	88.759%	2	−23.52
AKT2	481/481	472	98.129%	6	−17.09

### Risperidone Inhibited the Adipocytokine Signaling Pathway and Might Inhibit the Lipid β-Oxidation Protein CPT1A *in vitro*

**Figure 7:**
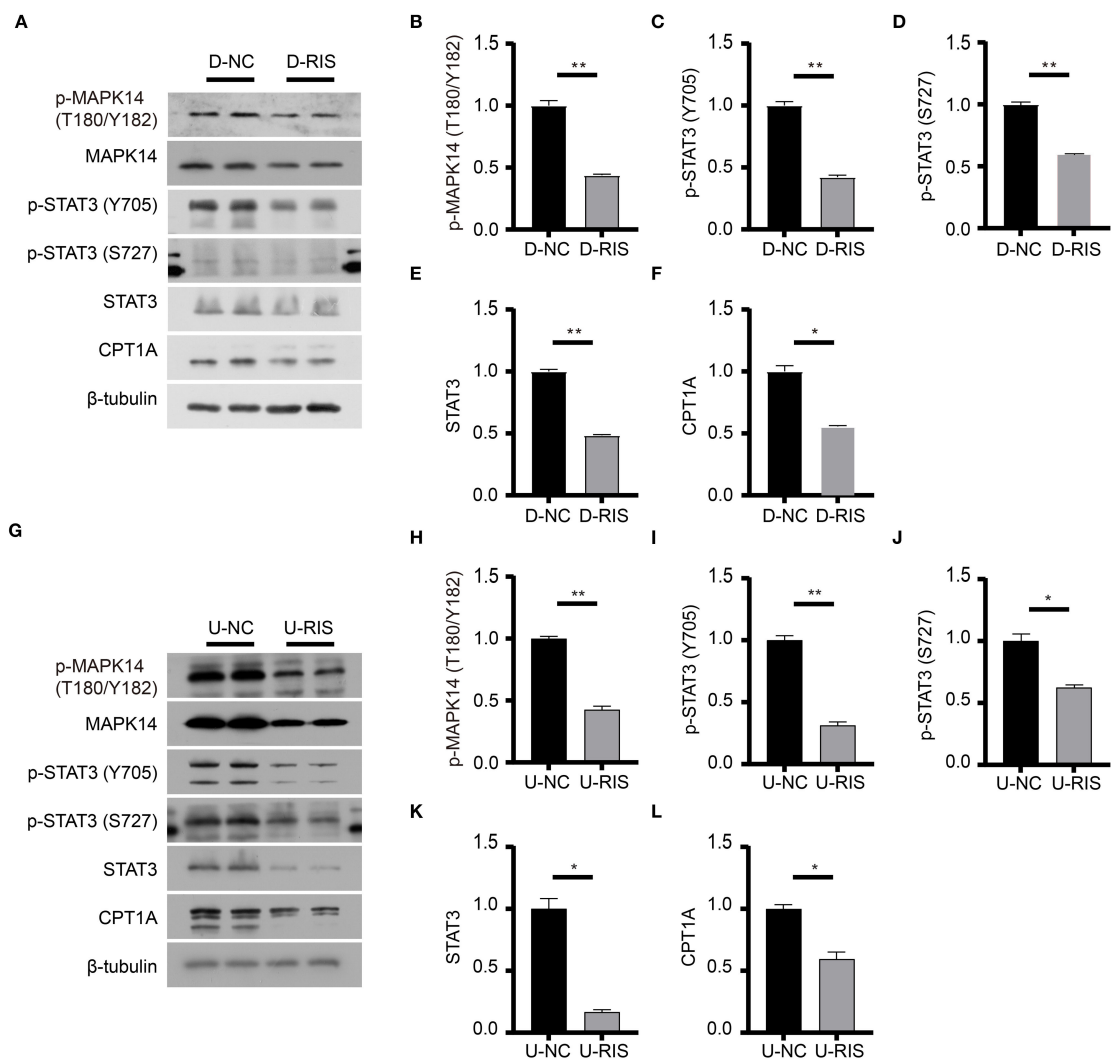
Immunoblotting analysis of effective factors in the adipocytokine signaling pathway. **(A)** Expression levels of effective factors in the adipocytokine signaling pathway in differentiated 3T3-L1 cells after treatment with DMSO or risperidone. **(B–F)** Gray degree analysis shows the expression level of effective factors in **(A)**. **(G)** Expression levels of effective factors in the adipocytokine signaling pathway in undifferentiated 3T3-L1 cells after treatment with DMSO or risperidone. **(H–L)** Gray degree analysis shows the expression level of effective factors in **(G)**. Values are expressed as the mean ± SEM (*n* = 2), *P < 0.05, **P < 0.01; ns, no significant difference; SEM, standard error of mean; D-NC, differentiated 3T3-L1 cells treated with DMSO; D-RIS, differentiated 3T3-L1 cells treated with risperidone; U-NC, undifferentiated 3T3-L1 cells treated with DMSO; U-RIS, undifferentiated 3T3-L1 treated with risperidone.

**Figure 8:**
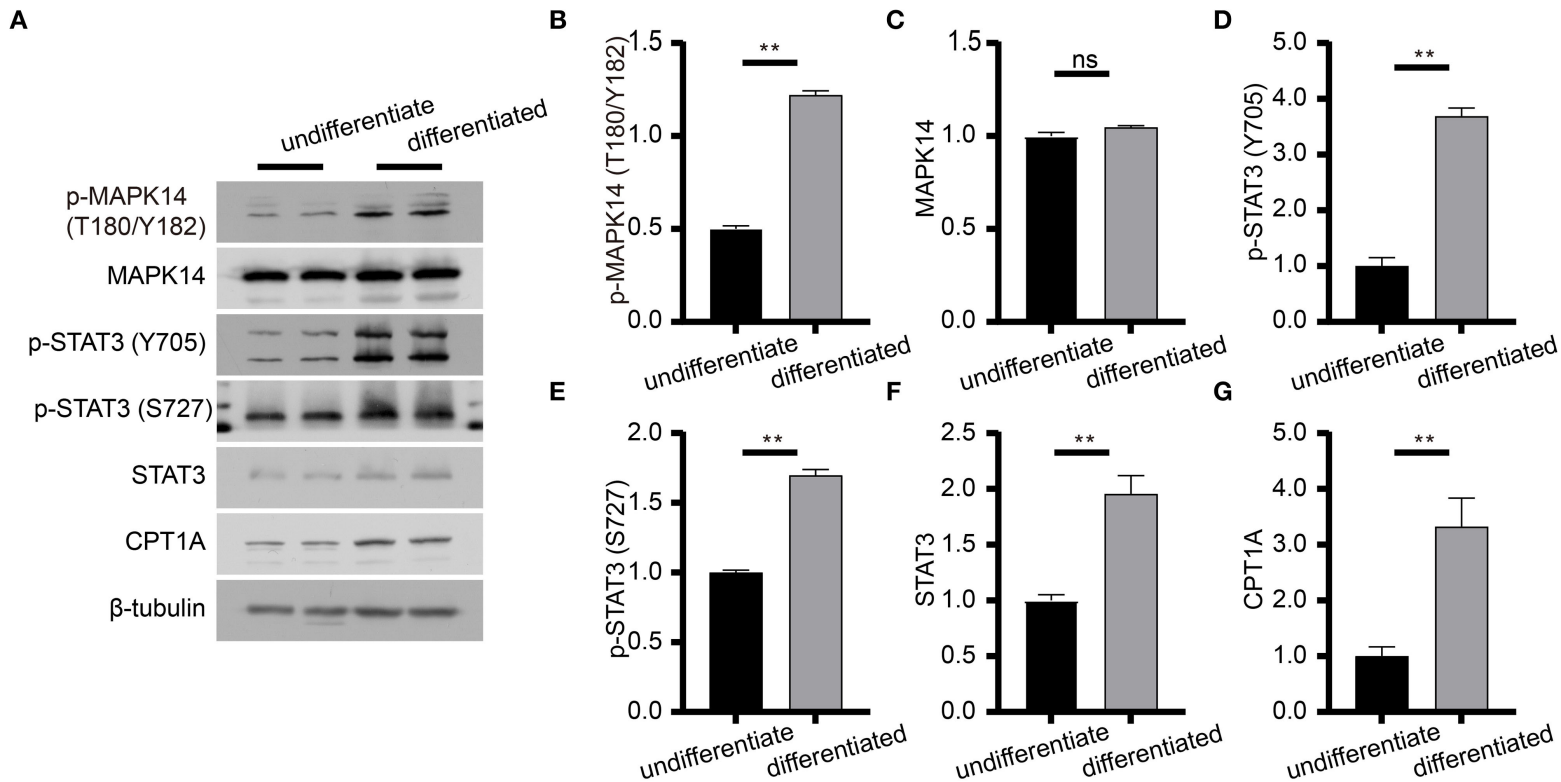
Immunoblot analysis of proteins in the adipocytokine signaling pathway. **(A)** Expression levels of effective factors in the adipocytokine signaling pathway in differentiated and undifferentiated 3T3-L1 cells. **(B–G)** Gray degree analysis shows the expression level of effective factors in **(A)**. Values are expressed as the mean ± SEM (*n* = 2), **P < 0.01; ns, no significant difference; SEM, standard error of mean.

To determine the effect of risperidone on the output of adipocytokine signaling pathway, we detected the expression levels of CPT1A and STAT3, which were crucial downstream effective factors of MAPK8 that play important roles in long-chain fatty acid β-oxidation and hepatic triglyceride metabolism. Our immunoblotting results showed that risperidone not only downregulated CPT1A and STAT3 expressions, it also decreased MAPK14 and STAT3 phosphorylation in differentiated and undifferentiated 3T3-L1 cells after DMSO or risperidone treatments (Figures 7A–L), indicating that risperidone inhibited signal transduction. As we know, lipid metabolisms, including lipogenesis and lipolysis, were more activated in adipocytes than in preadipocytes. To better understand the specificity of the role of risperidone in the adipocytokine signaling pathway and long-chain fatty acid β-oxidation, we performed the same validation and analysis on undifferentiated and differentiated 3T3-L1 cells. The results showed that the expression and corresponding phosphorylation levels of MAPK14, STAT3, and CPT1A were upregulated when the cells differentiated into adipocytes (Figures 8A–G).

## Discussion

To the best of our knowledge, we are the first to discover the mechanisms of risperidone-induced lipid accumulation using multiple crossover analyses, including network pharmacology, transcriptomics, and disease databases. Furthermore, we validated the candidate pathway and the adipocytokine signaling pathway and established a series of binding models by molecular docking to elucidate the mechanisms of risperidone-induced weight gain.

In our study, risperidone directly promoted adipocyte differentiation and lipid accumulation in both 3T3-L1 cells and AMSCs. Furthermore, the mRNA expression levels of *Apn, Rac1*, and *Fasn* were upregulated, while *Pparg* was not significantly changed. These cellular results indicate that risperidone treatment may directly affect adipose tissue independent of the central nervous system and food intake, which is consistent with previous studies (25, 27). The lack of significant upregulation of the mRNA expression levels of *Pparg* may be the result of feedback regulation, as increased Pparg protein expression was observed after risperidone treatment (Supplementary Figures 3A,B). Through network pharmacology analysis, we identified risperidone binding targets and found that these proteins have important biological functions, such as metabolic processes and responses to stimuli. In addition, these targets were involved in the PPAR signaling pathway and adipocytokine signaling pathway, which modulate multiple biological processes within cells, including lipid and glucose metabolism and energy balance (35). The above results suggested that risperidone may affect adipogenesis and adipocyte differentiation through the PPAR signaling pathway and adipocytokine signaling pathway.

After transcriptomic analysis, risperidone affected the transcription of related genes in the adipocytokine signaling pathways, as predicted by network pharmacology analysis. Among the downregulated genes, *STAT3* and *CPT1A* are closely associated with β-oxidation of long-chain fatty acids (36, 37), indicating that risperidone inhibits lipolysis. Furthermore, an inactive adipocytokine signaling pathway was associated with insulin resistance, increased food intake, and reduced energy expenditure, which may explain risperidone-induced weight gain due to hyperphagia (21).

We analyzed the top 3 proteins (AKT2, STAT3, and IKBKB) in lipid metabolism from the protein-protein interaction and KEGG enrichment analyses and the top 5 proteins (MAPK8, MDM2, MMP2, GSK3B, and CASP3) in intersection analysis with tight junctions (Supplementary Figure 4). In the KEGG analysis, MAPK8, AKT2, STAT3, and IKBKB were enriched in the adipocytokine signaling pathway, located downstream of the leptin receptor, and strongly related to adipocyte volume and number. Other proteins from these two analyses were shown to be in pathways related to glycolipid metabolism as well as endocrine regulation, which are closely associated with lipogenesis, indicating that proteins have a synergistic effect on lipid accumulation to a certain extent. Synergy among these proteins has also been supported. For example, GSK3β expression promotes ESCC cell progression through STAT3 (38), and GSK3β inhibition could block STAT3 signaling, reducing pro-inflammatory responses (39). STAT3 activation could lead to MMP2 expression through MAPK8, affecting cell migration and viability (40, 41). In addition, the stability of crucial factors in regulatory pathways may affect signal transduction. IKBKB could promote the stability of MDM2 (42), which can interact with MAPSK3 to promote STAT3 degradation (43). In conclusion, the proteins we screened have critical functions in cellular biological processes and lipid metabolism and may modulate the adipocytokine signaling pathway in a synergistic mode.

The β-oxidation of long-chain fatty acids is the main source of cellular energy and occurs in both mitochondria and peroxisomes (44). Mitochondria catalyze the β-oxidation of the bulk of short-, medium-, and long-chain fatty acids derived from diet, and this pathway constitutes the major process by which fatty acids are oxidized to generate energy. CPT1A acts as a catalytic enzyme by catalyzing the acyl transfer of long-chain fatty acid-CoA conjugations onto carnitine, which is required for mitochondrial uptake of long-chain fatty acids and subsequent β-oxidation in mitochondria (45). STAT3 is a nuclear transcription factor that is phosphorylated and activated by JAK2 (tyrosine-protein kinase JAK2) to form an activated homologous dimer that enters the nucleus to activate CPT1A transcription. Furthermore, STAT3 is required for leptin signaling transduction and activation (46). Our immunoblotting results showed that risperidone decreased STAT3 and CPT1A expression as well as their phosphorylation levels in differentiated and undifferentiated 3T3-L1 cells, which was not an indirect result of risperidone-induced cell differentiation (Figures 7A,H). These results suggest that risperidone can specifically inhibit the STAT3-CPT1A axis.

Our intersection analysis (Figure 4A) indicated that MAPK8, AKT2, RXRA, and RXRB were high-confidence candidates that also belong to the adipocytokine signaling pathway upstream of STAT3. In addition, MAPK14 is an upstream protein of the STAT3-CPT1A signaling pathway among the top 10 targets with the highest *Z*-score predicted by network pharmacology. Through molecular docking analyses (Figure 5) and immunoblotting validation (Figures 6, 7), it was concluded that risperidone inhibited MAPK14 and STAT3 phosphorylation and their downstream protein CPT1A by downregulating MAPK14, MAPK8, RXRA, and RXRB, thereby inhibiting the β-oxidation of fatty acids. It has been reported that MAPK14 is negatively correlated with lipid accumulation from MAPK14 knockout mice with increased peripheral fat (47). RXRA and RXRB, members of the RXR family, can be activated by sterols and are involved in a series of biological processes in cells, such as cell differentiation and fatty acid oxidation (48, 49). PPARs are members of the nuclear hormone receptor superfamily and have been implicated in the regulation of lipid metabolism and adipocyte differentiation (35). To bind DNA and activate transcription, PPARs must form heterodimers with RXR (50). Furthermore, PPARA could induce CPT1A expression using a PPARA agonist (51, 52) or fasting, which may explain why risperidone induces lipogenesis. Taken together, risperidone promotes lipid accumulation by inhibiting fatty acid β-oxidation.

There were several limitations in our study. First, we may have missed some of the predicted targets due to insufficient databases of protein structures. Moreover, our results demonstrated that MAPK8, RXRA, RXRB, and AKT2 were high-confidence candidates and played important roles in the β-oxidation of long-chain fatty acids. However, given that drug-target binding does not necessarily lead to changes in target expression, our screening criteria may be too stringent; thus, other targets may be overlooked. In the future, we should not only consider the intersection of these four targets but also pay attention to the 73 targets that are associated with obesity but do not change their expression levels.

In summary, the adipocytokine signaling pathway, as a novel signaling pathway, plays an important role in risperidone-induced lipogenesis. In addition, these findings could provide a more comprehensive understanding of the action of risperidone and a new administration strategy for risperidone-induced weight gain.

## Data Availability Statement

The datasets presented in this study can be found in online repositories. The names of the repository/repositories and accession number(s) can be found below: https://www.ncbi.nlm.nih.gov/geo/query/acc.cgi?acc=GSE198053.

## Author Contributions

W-DL and SL were responsible for the study concept and design, drafting of the manuscript, and study supervision. YF performed the experiments and prepared the initial draft of the manuscript. KY, YH, and YZ performed the experiments. All authors have contributed to and approved the final manuscript.

## Funding

This study was supported by the National Natural Science Foundation of China (92046014, 81801323), Beijing-Tianjin-Hebei Jointed Research Program (19JCZDJC64700), and National Key R&D Program of China (2017YFC1001900).

## Conflict of Interest

The authors declare that the research was conducted in the absence of any commercial or financial relationships that could be construed as a potential conflict of interest.

## Publisher's Note

All claims expressed in this article are solely those of the authors and do not necessarily represent those of their affiliated organizations, or those of the publisher, the editors and the reviewers. Any product that may be evaluated in this article, or claim that may be made by its manufacturer, is not guaranteed or endorsed by the publisher.
