# Supplementary material for: Deciphering Risperidone-Induced Lipogenesis by Network Pharmacology and Molecular Validation
Source: Front Psychiatry. 2022 Apr 18;13:870742. doi: 10.3389/fpsyt.2022.870742 (PMC9058120; doi:10.3389/fpsyt.2022.870742)
Supplement: Supplementary file 1 [file Data_Sheet_1.PDF]

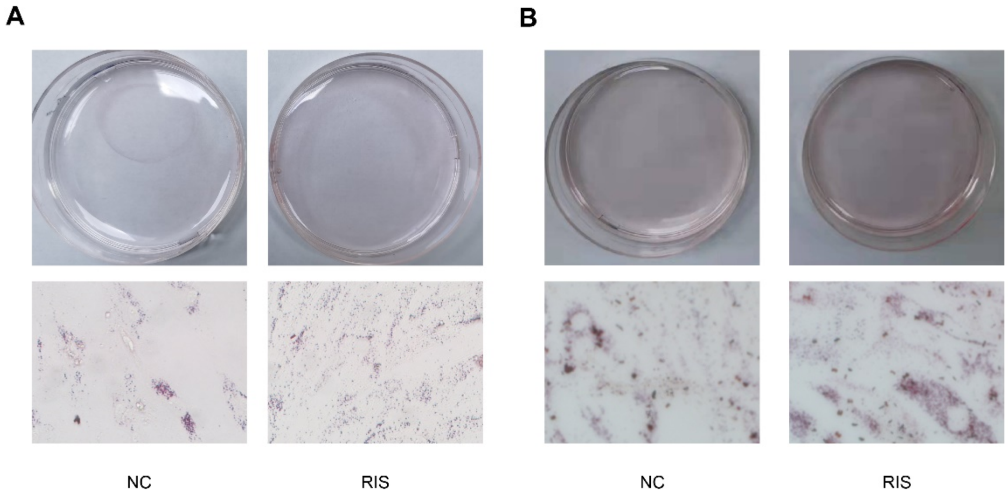

Figure S1 Risperidone promotes lipid accumulation and differentiation in AMSC. Oil red-O staining of **(A)** Undifferentiated AMSC and **(B)** differentiated AMSC treated by DMSO or risperidone (100 μM).

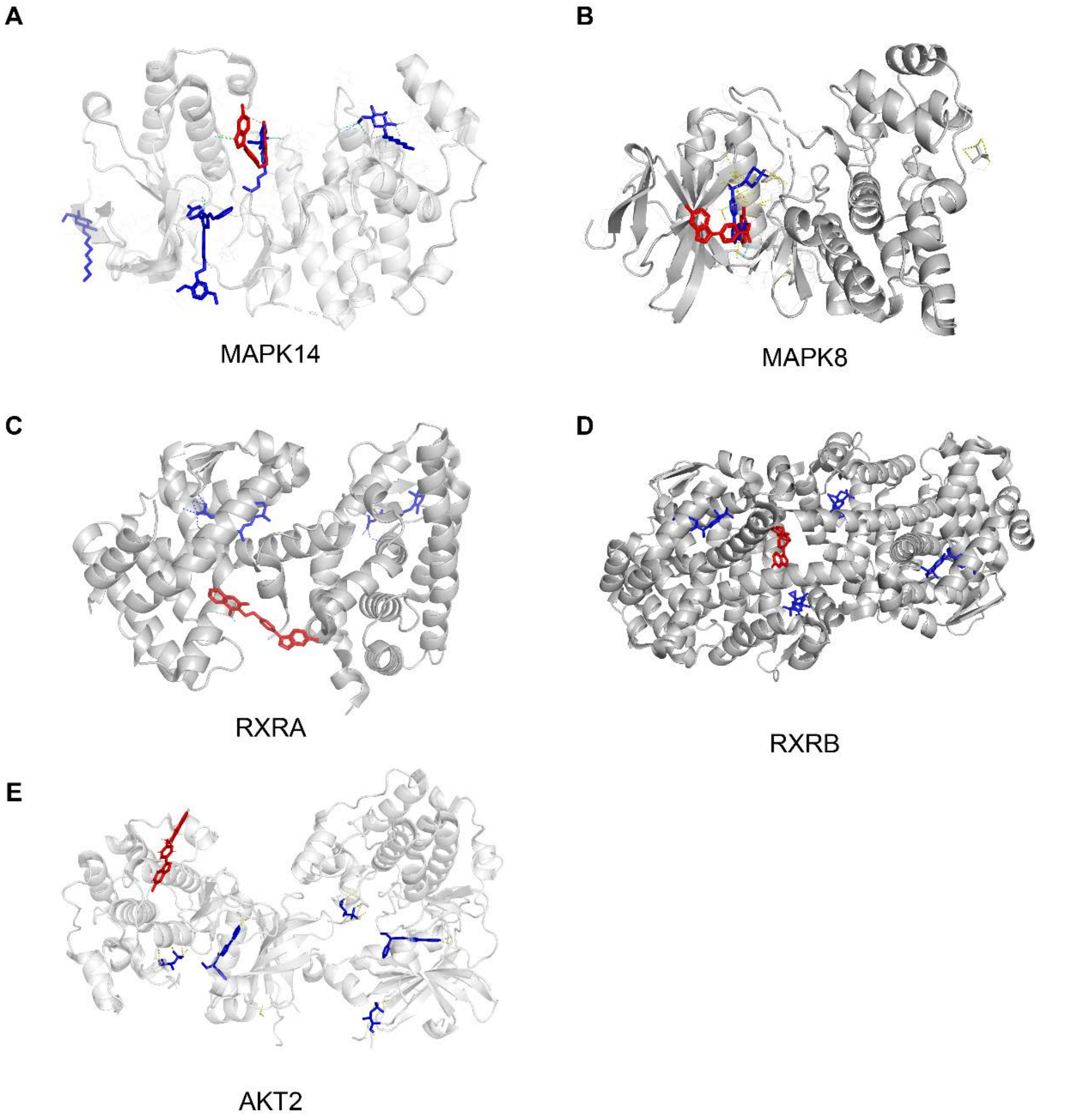

Figure S2 Docking comparisons between risperidone and ligand of the crystal targets. (A) Ligand of MAPK14: Octyl beta-D-glucopyranoside (PCCID: 62852). (B) Ligand of MAPK8: 4-Phenyl-7-azaindoles (PCCID: 354254). (C) Ligand of RXRA: 3-(5-(3,5-Bis(trifluoromethyl)phenyl)-4-phenyloxazol-2-yl) propanoic acid (PCCID: 155491000). (D) Ligand of: 6-(1-(3,5,5,8,8-Pentamethyl-5,6,7,8-tetrahydronaphthalen-2-yl) cyclopropyl) nicotinic acid (PCCID: 3922). (E) Ligand of: N-[(1s)-2-Amino-1-Phenylethyl]-5-(1h-Pyrrolo[2,3-B] pyridin-4-Yl) thiophene-2-Carboxamide (PCCID: 24963048). Ligands were shown as blue sticks, risperidone was shown as red sticks.

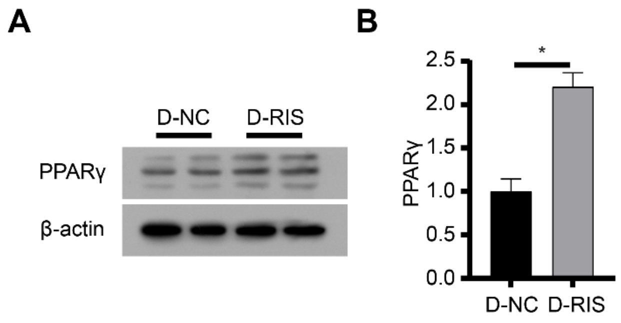

Figure S3 Risperidone promotes the expression of PPARG in differentiated 3T3L1. (A) Expression level of PPARG in differentiated 3T3-L1. **(B)** Gray degree analysis show the expression level of effective factors in **(A)**. Values are expressed as the mean ± SEM (n=2), *$P < 0.05$. SEM: Standard Error of Mean. D-NC: differentiated 3T3-L1 treated with DMSO. D-RIS: differentiated 3T3-L1 treated with Risperidone.

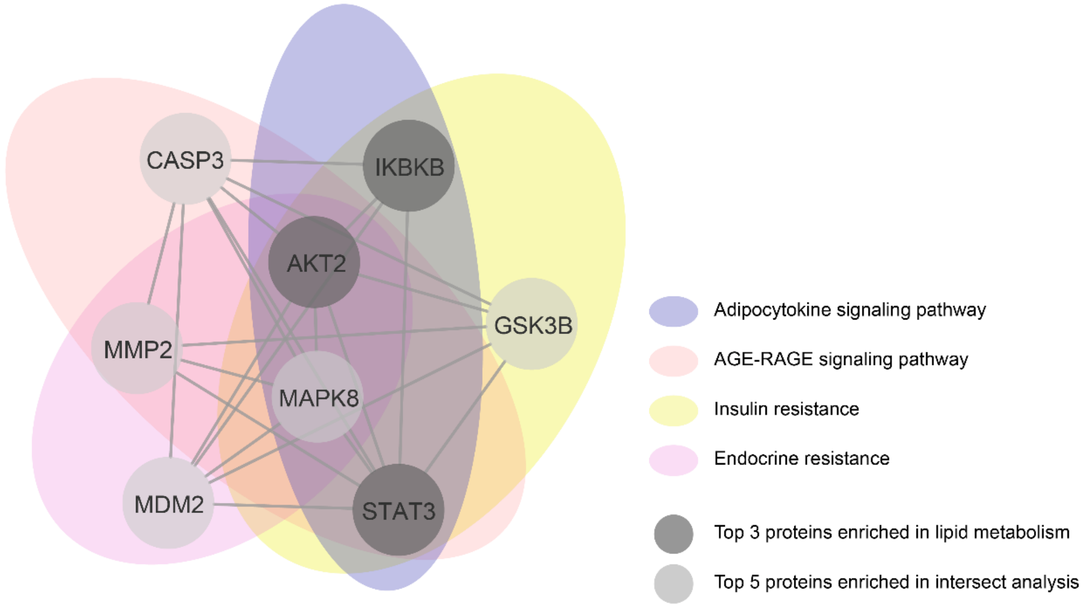

Figure S4 Protein-Protein Interaction analysis of the top three proteins involved in metabolic and lipid beta-oxidation related genes and top five proteins in obesity-related genes.
