# Supplementary material for: Deciphering Risperidone-Induced Lipogenesis by Network Pharmacology and Molecular Validation
Source: Front Psychiatry. 2022 Apr 18;13:870742. doi: 10.3389/fpsyt.2022.870742 (PMC9058120; doi:10.3389/fpsyt.2022.870742)
Supplement: Supplementary file 2 [file Table_1.docx]

**Table S1 236 predicted targets by network pharmacology analysis**

| Name | zscore |
| --- | --- |
| cAMP-specific 3,5-cyclic phosphodiesterase 4B | 1.48482 |
| Carbonic anhydrase 2 | 1.15191 |
| SPARC | 0.645754 |
| ADP-ribose pyrophosphatase, mitochondrial | 0.440217 |
| P-selectin | 0.308287 |
| Calmodulin | 0.3581 |
| Cell division protein kinase 2 | 0.539259 |
| Intercellular adhesion molecule 2 | 0.252484 |
| Complement factor B | 1.81668 |
| Alpha-amylase 1 | 2.01835 |
| Eosinophil lysophospholipase | -0.54411 |
| cAMP-specific 3,5-cyclic phosphodiesterase 4D | 1.15712 |
| Proto-oncogene serine/threonine-protein kinase Pim-1 | 1.13676 |
| Neutrophil gelatinase-associated lipocalin | 0.793519 |
| Cyclin-A2 | 0.707463 |
| Purine nucleoside phosphorylase | 0.644513 |
| S-methyl-5-thioadenosine phosphorylase | -1.43541 |
| Serine/threonine-protein kinase Chk1 | 0.587718 |
| Angiogenin | 0.007514 |
| Heat shock protein HSP 90-alpha | 2.10075 |
| Inositol monophosphatase | 3.89429 |
| NONE | -0.01925 |
| Activated CDC42 kinase 1 | 1.48051 |
| Glucosylceramidase | 1.36972 |
| Glycogen synthase kinase-3 beta | 1.30622 |
| Superoxide dismutase [Mn], mitochondrial | 1.16323 |
| 3-hydroxy-3-methylglutaryl-coenzyme A reductase | 0.913111 |
| Hexokinase-1 | 0.743915 |
| Phenylalanine-4-hydroxylase | 1.25709 |
| Pancreatic alpha-amylase | 1.15969 |
| Interferon-stimulated gene 20 kDa protein | 0.563155 |
| Adenosylhomocysteinase | 0.652227 |
| Tyrosine-protein phosphatase non-receptor type 1 | 1.06132 |
| Zinc-alpha-2-glycoprotein | -0.43252 |
| Androgen receptor | 0.898639 |
| Deoxycytidine kinase | 0.88621 |
| Cytidine deaminase | 0.594834 |
| Urokinase-type plasminogen activator | 0.694908 |
| Lanosterol synthase | 0.312782 |
| Copper transport protein ATOX1 | 0.63544 |
| Ras-related protein Rab-5A | 2.14162 |
| Inosine-5-monophosphate dehydrogenase 2 | -0.16013 |
| Salivary alpha-amylase | 2.15869 |
| GTP-binding protein Rheb | 0.364364 |
| Kinesin-like protein KIF11 | -0.09424 |
| Heat shock protein homolog SSE1 | 1.34911 |
| Placenta growth factor | -0.18152 |
| Catenin alpha-1 | -0.20993 |
| Glutathione S-transferase theta-2 | 2.79348 |
| Heat shock cognate 71 kDa protein | 0.021431 |
| Transforming protein RhoA | -0.02482 |
| Glycogen phosphorylase, liver form | 1.20576 |
| Prothrombin | 0.152154 |
| Aldose reductase | 0.901748 |
| Tryptophan 5-hydroxylase 1 | 2.00242 |
| Ras-related protein Rab-11A | 1.10462 |
| Cyclin-dependent kinase 5 activator 1 | 1.35763 |
| Galactokinase | -0.22456 |
| ADP-ribosyl cyclase 2 | 0.884617 |
| Phosphoenolpyruvate carboxykinase, cytosolic [GTP] | 0.397402 |
| Lithostathine-1-alpha | -0.42686 |
| Uridine 5-monophosphate synthase | 0.999877 |
| Interleukin-2 | 1.03959 |
| Galectin-2 | 0.901767 |
| Beta-hexosaminidase subunit beta | 1.03071 |
| Bis(5-adenosyl)-triphosphatase | 1.1901 |
| Cytochrome P450 2C9 | 0.537179 |
| Eosinophil cationic protein | 0.620232 |
| Carbonyl reductase [NADPH] 1 | -0.8566 |
| Nitric oxide synthase, inducible | 0.880714 |
| Stromelysin-1 | 0.359529 |
| Arylsulfatase A | 0.59719 |
| Fructose-bisphosphate aldolase A | 0.400795 |
| Dipeptidyl peptidase 4 | -0.80481 |
| Serine/threonine-protein kinase PAK 6 | 0.038355 |
| Galactosylgalactosylxylosylprotein 3-beta-glucuronosyltransferase 1 | 0.605625 |
| Prostatic acid phosphatase | 0.310015 |
| Leukocyte elastase | 0.112168 |
| NAD(P)H dehydrogenase [quinone] 1 | -0.01654 |
| Serum albumin | -0.34969 |
| Triggering receptor expressed on myeloid cells 1 | -0.30484 |
| Protein-glutamine gamma-glutamyltransferase E | -0.07756 |
| C-C motif chemokine 5 | -0.11678 |
| 3-phosphoinositide-dependent protein kinase 1 | -1.23597 |
| Rho GTPase-activating protein 1 | 0.02385 |
| Glucosamine-6-phosphate isomerase | 1.02819 |
| Endoplasmic reticulum mannosyl-oligosaccharide 1,2-alpha-mannosidase | 1.01418 |
| Uridine-cytidine kinase 2 | -0.52361 |
| Glutathione reductase, mitochondrial | -0.2427 |
| S-adenosylmethionine decarboxylase proenzyme | -1.15897 |
| Mast/stem cell growth factor receptor | 0.690849 |
| Inosine-5-monophosphate dehydrogenase 1 | 0.292191 |
| Cathepsin S | 0.558479 |
| Beta-secretase 1 | 0.841435 |
| Coagulation factor X | 2.18333 |
| Thymidine kinase, cytosolic | 0.790788 |
| Tyrosine-protein kinase JAK2 | -0.09121 |
| Spermidine synthase | 0.329563 |
| Hydroxyacylglutathione hydrolase, mitochondrial | 0.324288 |
| cAMP-dependent protein kinase catalytic subunit alpha | 0.397521 |
| GTPase HRas | 1.27912 |
| cGMP-specific 3,5-cyclic phosphodiesterase | 0.120388 |
| Glucose-6-phosphate isomerase | -0.33418 |
| Peptidyl-prolyl cis-trans isomerase FKBP1A | -0.06466 |
| Neutrophil collagenase | 0.402731 |
| Adenosine kinase | 0.300998 |
| Vascular endothelial growth factor receptor 2 | 0.220677 |
| Dihydroorotate dehydrogenase, mitochondrial | -1.22557 |
| Coagulation factor VII | -0.49855 |
| Early endosome antigen 1 | 2.32677 |
| Proto-oncogene tyrosine-protein kinase Src | -1.69548 |
| Chitotriosidase-1 | 1.39919 |
| Ras-related protein Rab-9 | 2.08648 |
| Mitogen-activated protein kinase 14 | 0.229392 |
| RAC-beta serine/threonine-protein kinase | 0.388699 |
| Cystathionine beta-synthase | -0.37585 |
| Baculoviral IAP repeat-containing protein 4 | -0.00893 |
| Neprilysin | 0.707026 |
| Aldo-keto reductase family 1 member C3 | -0.52434 |
| Beta-hexosaminidase beta chain | 0.242438 |
| Adenine phosphoribosyltransferase | -0.35065 |
| Baculoviral IAP repeat-containing protein 7 | 1.26533 |
| T-cell surface glycoprotein CD1a | -0.66984 |
| Proto-oncogene tyrosine-protein kinase ABL1 | 0.642566 |
| GTP-binding nuclear protein Ran | 1.20049 |
| Galectin-3 | -0.21091 |
| Dual specificity protein kinase CLK1 | -0.53959 |
| Tryptophanyl-tRNA synthetase, cytoplasmic | 1.66112 |
| Farnesyl pyrophosphate synthetase | -0.12445 |
| Histidine triad nucleotide-binding protein 1 | 0.596949 |
| Dihydrofolate reductase | -0.81968 |
| 5(3)-deoxyribonucleotidase, mitochondrial | 0.660763 |
| Hydroxyacyl-coenzyme A dehydrogenase, mitochondrial | 0.669775 |
| Ferrochelatase, mitochondrial | -0.0065 |
| Glycolipid transfer protein | 1.02447 |
| Gastrotropin | -1.01276 |
| Progesterone receptor | 0.100533 |
| Mineralocorticoid receptor | -1.2771 |
| Corticosteroid 11-beta-dehydrogenase isozyme 1 | -0.72653 |
| L-serine dehydratase | -1.11939 |
| Alpha-1-antitrypsin | -1.82081 |
| Phospholipase A2, membrane associated | -0.25394 |
| Ornithine carbamoyltransferase, mitochondrial | -0.21777 |
| Coagulation factor XI | 0.144742 |
| Nicotinamide mononucleotide adenylyltransferase 1 | -1.23931 |
| Trifunctional purine biosynthetic protein adenosine-3 | -0.32055 |
| Histone deacetylase 8 | -2.18056 |
| C-1-tetrahydrofolate synthase, cytoplasmic | -2.44218 |
| Ephrin type-A receptor 2 | -1.33581 |
| Glutathione S-transferase omega-1 | -0.10003 |
| Cathepsin K | 0.533713 |
| Interstitial collagenase | -0.19319 |
| RAF proto-oncogene serine/threonine-protein kinase | 0.567889 |
| Proto-oncogene tyrosine-protein kinase LCK | 0.028097 |
| Estrogen sulfotransferase | -0.28292 |
| Serine--pyruvate aminotransferase | -1.47175 |
| Histo-blood group ABO system transferase | -1.3843 |
| Glucocorticoid receptor | -0.13479 |
| Glutathione-requiring prostaglandin D synthase | -0.50677 |
| Inositol-trisphosphate 3-kinase A | 0.780385 |
| Nicotinamide mononucleotide adenylyltransferase 3 | 0.177135 |
| Renin | 0.866913 |
| Bifunctional purine biosynthesis protein PURH | 0.046363 |
| Glutathione S-transferase Mu 1 | 0.61575 |
| Methionine aminopeptidase 2 | 0.783005 |
| Glutathione S-transferase A1 | 0.241691 |
| Cell division control protein 42 homolog | -0.86668 |
| GMP reductase 1 | 0.647226 |
| Betaine--homocysteine S-methyltransferase 1 | -0.69728 |
| Matrix metalloproteinase-16 | -0.74436 |
| Glutathione S-transferase A3 | -0.15681 |
| Protein-L-isoaspartate(D-aspartate) O-methyltransferase | 0.18191 |
| Hypoxanthine-guanine phosphoribosyltransferase | -0.25514 |
| Non-secretory ribonuclease | -0.20467 |
| Bifunctional heparan sulfate N-deacetylase/N-sulfotransferase 1 | 0.008048 |
| Caspase-3 | 0.362344 |
| Mitogen-activated protein kinase 10 | 0.064638 |
| Pyruvate kinase isozymes R/L | 0.455772 |
| Fibrinogen gamma chain | 0.93332 |
| ADP-ribosylation factor-like protein 5A | -0.31092 |
| Matrix metalloproteinase-9 | -1.12382 |
| Transforming growth factor beta-2 | -0.37421 |
| Glutathione S-transferase Mu 2 | -0.45341 |
| Thymidylate kinase | -0.58433 |
| Histone-lysine N-methyltransferase, H3 lysine-79 specific | 0.483937 |
| Ras-related protein Rap-2a | 0.521617 |
| UDP-glucose 4-epimerase | -0.29952 |
| Eukaryotic translation initiation factor 4E | -1.11348 |
| 3 histone mRNA exonuclease 1 | -0.35802 |
| Arginase-2, mitochondrial | -2.36036 |
| Maleylacetoacetate isomerase | -0.00722 |
| Sulfotransferase family cytosolic 2B member 1 | -1.62116 |
| Phenylethanolamine N-methyltransferase | 0.4968 |
| Deoxyuridine 5-triphosphate nucleotidohydrolase, mitochondrial | -0.41788 |
| Scavenger mRNA-decapping enzyme DcpS | 0.456807 |
| ADAM 33 | -0.33281 |
| Histone acetyltransferase PCAF | -1.05098 |
| Peptidyl-prolyl cis-trans isomerase FKBP1B | -0.57939 |
| GMP reductase 2 | -0.21364 |
| Quinone oxidoreductase | 0.82235 |
| E-selectin | -1.07894 |
| SEC14-like protein 2 | -0.03291 |
| ADP-ribosylation factor-like protein 5B | 0.15472 |
| Leukotriene A-4 hydrolase | -1.15998 |
| Histamine N-methyltransferase | -0.53845 |
| Sulfotransferase 1A1 | 0.468597 |
| UDP-N-acetylhexosamine pyrophosphorylase | 0.296061 |
| L-xylulose reductase | 0.167495 |
| Ras-related C3 botulinum toxin substrate 1 | -0.00169 |
| Riboflavin kinase | -0.20163 |
| Death-associated protein kinase 1 | -0.25951 |
| Hepatocyte growth factor receptor | -0.1655 |
| tRNA (cytosine-5-)-methyltransferase | -0.68336 |
| Caspase-1 | -0.12301 |
| Ornithine aminotransferase, mitochondrial | -2.21556 |
| Glutathione S-transferase P | 0.558963 |
| Platelet glycoprotein Ib alpha chain | -2.62656 |
| NAD-dependent malic enzyme, mitochondrial | -0.7049 |
| Rho-related GTP-binding protein RhoE | -0.9671 |
| CD209 antigen | -2.53721 |
| Tryptase beta-2 | -0.68503 |
| Serine/threonine-protein phosphatase PP1-gamma catalytic subunit | -0.49836 |
| Histone-lysine N-methyltransferase SETD8 | 0.470678 |
| Bile salt sulfotransferase | -0.6433 |
| Tyrosine-protein kinase BTK | -0.29358 |
| Apoptotic protease-activating factor 1 | -0.85912 |
| Isovaleryl-CoA dehydrogenase, mitochondrial | 0.223438 |
| Antigen peptide transporter 1 | -0.93167 |
| Nucleoside diphosphate kinase B | -1.68137 |
| RAC-alpha serine/threonine-protein kinase | -0.99942 |
| Sepiapterin reductase | 0.129827 |
| NAD-dependent deacetylase sirtuin-5 | -0.15348 |
| FK506-binding protein 3 | -1.8442 |
| Adenylate kinase isoenzyme 1 | -1.64507 |
| Amine oxidase [flavin-containing] B | 0.287844 |
| Estradiol 17-beta-dehydrogenase 1 | -0.20897 |
